# Pharmacological methods for ovarian function and fertility preservation in women with cancer: A literature review

**DOI:** 10.32604/or.2024.049743

**Published:** 2024-07-17

**Authors:** ANA S. CVETANOVIC, MATTEO LAMBERTINI, KEVIN PUNIE, GORANA G. MATOVINA BRKO, NIKOLA D. ZIVKOVIC, MAJA J. POPOVIC, MARIJANA M. MILOVIC KOVACEVIC, LAZAR S. POPOVIC

**Affiliations:** 1 Department for Oncology, Medical Faculty Nis, University of Nis, Nis, 18000, Serbia; 2 Clinic of Oncology, University Clinical Centre Nis, Nis, 18000, Serbia; 3 Department of Internal Medicine and Medical Sciences (DiMI), School of Medicine, University of Genova, Genova, 16100, Italy; 4 Department of Medical Oncology, U.O.C Clinica di Oncologia Medica, IRCCS Ospedale Policlinico San Martino, Genova, 16100, Italy; 5 Department of General Medical Oncology and Multidisciplinary Breast Centre, Leuven Cancer Institute, University Hospitals Leuven, Leuven, 3001, Belgium; 6 Department of Medical Oncology, Oncology Institute of Vojvodina, Sremska Kamenica, 21208, Serbia; 7 Department for Pathology, Medical Faculty Nis, University of Nis, Nis, 18000, Serbia; 8 Center for Pathology, University Clinical Centre Nis, Nis, 18000, Serbia; 9 Faculty of Medicine, University of Novi Sad, Novi Sad, 21000, Serbia; 10 Department of Medical Oncology, Institute of Oncology and Radiology Serbia, Belgrade, 11000, Serbia

**Keywords:** Gonadotropin-releasing hormone agonists (GnRHa), Fertility preservation, Premature ovarian insufficiency (POI), Premenopausal patients

## Abstract

Oncofertility is an extremely significant topic that is increasingly being discussed owing to increased evidence indicating that fertility preservation does not affect the treatment outcomes of patients with cancer but significantly contributes to preserving life quality. The effect of chemotherapy can range from minimal effects to complete ovarian atrophy. Limited data are available on the effects of monoclonal antibodies and targeted therapies on the ovaries and fertility. Temporary ovarian suppression by administering a gonadotropin-releasing hormone agonist (GnRHa) during chemotherapy decreases the gonadotoxic effect of chemotherapy, thereby diminishing the chance of developing premature ovarian insufficiency (POI). At present, the concomitant administration of GnRH analogs during chemotherapy is the only accepted pharmacological method for preserving ovarian function. Notably, most randomized studies on the effectiveness of luteinizing hormone-releasing hormone agonists during chemotherapy in preventing POI have been conducted in women with breast cancer, with a considerably small number of studies on patients with hematological malignancies. Furthermore, most randomized controlled trials on breast cancer have revealed a decrease in treatment-induced POI risk, regardless of the hormone receptor status. In addition, studies on hematological malignancies have yielded negative results; nevertheless, the findings must be interpreted with caution owing to numerous limitations. Current guidelines from the American Society of Clinical Oncology and ESMO Clinical Practice Guidelines recommend sperm, oocyte, and embryo cryopreservation as a standard practice and only offering GnRHa to patients when proven fertility preservation methods are not feasible. In this manuscript, we present a comprehensive literature overview on the application of ovarian suppression with GnRHa during chemotherapy in patients with cancer by addressing preclinical and clinical data, as well as future perspectives in this field that upcoming research should focus on.

## Introduction

With the introduction of new cancer therapy options, the treatment outcomes of women with different malignancy types have substantially improved. In addition to an extended life expectancy, considering the quality of life of patients with cancer is vital. Preserving ovarian function and fertility is an essential aspect to consider when treating premenopausal women with cancer. Breast cancer, hematological malignancies, and ovarian cancer are some of the malignancies with treatments most associated with gonadal dysfunction (e.g., premature ovarian insufficiency [POI]). POI can lead to infertility and premature menopause with several symptoms, including weakness, depression, hot flashes, vaginal dryness, changes in hair quality, and sexual dysfunction; all these symptoms significantly affect the quality of life [1–3].

Before starting therapy with potential gonadotoxic effects, clinicians must discuss all possible fertility preservation methods with the patient immediately after diagnosis because some of these procedures are time-consuming and may lead to treatment delays if not properly organized. Oocyte, embryo, or ovarian tissue cryopreservation is a widely accepted standard fertility preservation strategy; however, it is exclusively available for women up to 36 years of age. Oocyte or embryo cryopreservation takes at least 2–3 weeks; therefore, that is the reason why it is not suitable for all women, particularly for those with aggressive malignancies requiring urgent treatment [4–6].

At present, the use of a gonadotropin-releasing hormone agonist (GnRHa) at the same time as chemotherapy is the only available pharmacological method for preserving ovarian function [7]. Initial mice studies in the 1980s revealed using GnRh analogs protected the gonadal function of cyclophosphamide-treated male mice. In 1987, Waxman et al. published the first randomized study on the role of GnRH in fertility preservation during chemotherapy [8]. The current clinical guidelines of the American Society of Clinical Oncology (ASCO) and European Society for Medical Oncology (ESMO) recommend using GnRh analogs and chemotherapy simultaneously; however, the precise mechanism by which these agents preserve fertility remains unclear; furthermore, this fertility preservation method cannot replace proven methods such as cryopreservation [9]. In this review, we focus on the results of existing randomized controlled trials (RCTs) and meta-analyses that have investigated the use of GnRha for ovarian function and fertility preservation, the mechanisms of action of this strategy, and its limitations.

### Effect of oncological therapy on ovarian function and fertility

Despite the development of several innovative therapies, chemotherapy and radiotherapy remain the cornerstone of oncological therapy. The activity of these therapies on fertility depends on the agent type, dose, exposure duration, gonadal reserve, and patient’s age. The chances of restoring the menstrual cycle after chemotherapy-induced amenorrhea (cessation of menstruation) are higher in women who are under 40 years old than in older women. Furthermore, germline mutations could have a role. Studies have revealed that germline pathogenic mutations in BRCA genes are associated with decreased ovarian reserve; however, the data remain contradictory [10,11].

### Chemotherapy

Cytotoxic drugs disrupt the normal cell cycle, resulting in DNA damage and oxidative stress in both somatic and germ cells. When left unrepaired, double strand breaks in DNA lead to oocyte apoptosis, resulting in aneuploidy and early embryo death. The impact of chemotherapy can cause loss of primordial follicles and ovarian atrophy [12–14]. In the adult’s ovaries, most follicles are in the primordial stage. Follicle growth depends on the secretion of follicle stimulating hormone (FSH) et luteinizing hormone (LH) which activates proliferation of granulosa cells, theca cell differentiation, and steroidogenesis. Also, hormonal status, the level of FSH, and the level of inhibin B are very important for follicle growth as well as for the ovarian volume and the number of antral follicles (AFC). Anti-mullerian hormone (AMH) is secreted by granulosa cells and presents a biochemical marker of fertility and follicular reserve. In the adults with age AMH concentrations decline and correlate with ovarian response to FSH. All follicular stages and cell types are involved in the mechanisms underlying chemotherapy-induced POI. Owing to the apoptosis of growing follicles, the remaining follicles become activated and subsequently undergo apoptosis. The lower the ovarian reserve, the greater the effect of chemotherapy. Chemotherapy works in a way that induces DNA double stranded breaks (DSBs) damage. Ataxia telangiectasia mutated kinase is the main repair mechanism of DSB. If the repair mechanism fails, cell apoptosis occurs. The greatest impact is on the growing follicles and there is less influence on primordial follicles. Reduction of growing follicles results in decreased growth factors such as amph and that is followed by recruitment of actively growing follicles and decreased number of primordial follicle (PMF). In addition, stromal blood vessels undergo fibrosis, further contributing to ovarian damage. All these factors together contribute to POI, with the therapy type and patient’s age being the most significant risk factors. Therefore, pharmacological protection is challenging because it should decrease gonadotoxicity at different levels [15–18].

Alkylating agents such as cyclophosphamide are universally accepted for treating various malignancies; however, they exert the most significant gonadotoxic effect. In a meta-analysis, Zhao et al. reported that individuals who received cyclophosphamide had a significantly higher amenorrhea rate compared with those who did not receive cyclophosphamide. Clinical guidelines have presented the level of risk associated with specific regimens. High-risk regimens that lead to amenorrhea in more than 80% of patients include six cycles of cyclophosphamide/busulfan; cyclophosphamide, methotrexate, and fluorouracil (CMF); cyclophosphamide, epirubicin, and fluorouracil; cyclophosphamide, adriamycin, and fluorouracil (CAF/FAC); and Taxotere, adriamycin, and cyclophosphamide for women aged ≥40 years [19].

Limited data are available on the effect of monoclonal antibodies and targeted therapies on the ovaries and fertility [4,20,21]. Studies on the efficacy of trastuzumab, including ALLTO and APT, did not reveal the significant effect of anti-HER2 therapy on POI. Recently, studies have revealed the improved event-free survival of immune checkpoint inhibitors (ICIs) in triple-negative breast cancer patients; however, they also demonstrated that ICIs may result in primary and secondary hypogonadism and libido and sexual impairment. Furthermore, ICIs may result in thyroiditis, adrenalitis, and hypophysitis; these conditions may affect fertilization by impairing the hormonal regulation of the hypothalamic–pituitary–ovarian axis [22]. Winship et al. used mouse models to evaluate the effect of ICIs inhibiting programmed cell death protein ligand 1 and cytotoxic T lymphocyte-associated antigen 4 on the ovaries and discovered that ICIs diminish the ovarian follicular reserve and impair oocyte maturation and ovulation. However, the exact effect on gonadal function and ovarian reserve in women remains unknown [23]. In addition, preclinical research has revealed that PARP inhibitors such as olaparib, which have been approved for patients with pathogenic BRCA1/2 mutations, exert gonadotoxic effects. Because poly ADP ribose polymerase (PARP) inhibitors are also utilized for early-stage breast cancer, examining their effect on POI is vital [24].

### Effect of radiotherapy on ovarian function and fertility

Radiotherapy to the abdomen or the lesser pelvis forms a treatment portion for various malignancies in women, primarily gynecological cancers. Radiotherapy is frequently administered concomitantly with chemotherapy (usually platinum agents), primarily based on the fact that platinum agents lead to DNA damage and carry a moderate risk of amenorrhea [25]. The number of primordial follicles decreases significantly from birth to menopause. Ionizing radiation causes the damage to DNA of follicles which can lead to a decrease in ovarian reserve. Natural follicle reduction can be quickened by ionizing radiation which leads to reduced hormone production. Decreased levels of estrogen can cause uterine dysfunction and premature menopause. Primordial follicles are more resistant to radiation compared to growing follicles. Very important for ovarian failure is the age of the patient, radiation dose, chemotherapy agent, and field covered by radiation. A very low dose of radiation ≤2 Gy can destroy half of the immature human oocytes and a dose of 25–50 Gy can cause infertility in the majority of women older than 40 [26–30].

### Mechanism of action of GnRH analogs and their effect on fertility (preclinical data)

Drs. Guillemin and Schally identified the structure of GnRHa in 1977; they observed that it comprises 92 amino acids. It is produced in the hypothalamus and reaches the pituitary gland via the bloodstream. In the hypothalamus, GnRH can activate GnRH receptors to stimulate FSH and LH secretion [31,32].

GnRH analogs also act on GnRH receptors and initially lead to the release of substantial amounts of gonadotropins, subsequently desensitizing GnRH receptors (flare-up effect) after 3 weeks. Although the mechanism by which GnRH analogs contribute to ovarian function and potential fertility preservation remains unclear, several possibilities have been proposed [30].

The protective strategy of GnRh could be explained by direct and indirect effects. In the ovaries of adult women, more than 90% of the ovarian reserve comprises primordial follicles, and their development into Graafian follicles is an FSH-dependent process. Desensitization of the pituitary gland using GnRha and decreased FSH secretion decrease follicle maturation, resulting in a higher number of dormant follicles that are less sensitive to the effects of cytotoxic agents. However, this theory is controversial because primordial follicle recruitment is gonadotropin-independent and occurs irrespective of FSH levels; this is because FSH is only needed for the development of the late follicular. Nevertheless, suppressing FSH by using GnRHa may prevent damage to early-growing follicles. Decreased level of FSH inhibits the proliferation of follicular cells. In the absence of GnRHa therapy, an increase in FSH level appears because low levels of estrogen and FSH stimulate the proliferation of GC thus further increasing sensitivity to chemotherapy. So, GnRHa maintains FSH on a low level and protects growing follicles [33].

On the other hand, preclinical studies using rodent models have revealed that high estrogen levels in the body increase ovarian perfusion by affecting the endothelium of blood vessels. Furthermore, decreasing estrogen levels in the body decreases ovarian blood flow, with decreasing ovarian perfusion exerting a protective function against chemotherapy-induced ovarian fibrosis [34,35].

Besides estrogen, growing follicles also secrete anti-mullerian hormone (AMH), negatively regulating the recruitment of primordial follicles. After chemotherapy, AMH levels decrease owing to damage to the growing follicles; subsequently, primordial follicles are massively recruited into the growing group of follicles called the “burnout effect”. By reducing the prompt decrease of AMH level, GnRHa may reduce the burnout effect. Studies with rats showed that cyclophosphamide induced decreased the level of AMH and this effect is reduced by using GnRHa. GnRHa can regulate AMH levels during chemotherapy and protect ovarian function [35–38].

Sphingosine-1-phosphate (S1P) is a molecule with anti-apoptotic and angiogenesis role in ovaries. It is known that GnRHa increases the expression of S1P and reduces apoptosis of cells. S1P is also improving neoangiogenesis in ovarian cells and prevents oocyte death [33].

There is a broad range of preclinical data on alternative pharmacological methods of ovarian protection. The best-known mechanisms of cyclophosphamide and cisplatin effect are DNA damage and cell apoptosis. Different agents are investigated to prevent oocyte apoptosis like protein kinase inhibitors and inhibitors of ceramide pathways. Imatinib, a protein kinase inhibitor, showed inconclusive results in fertility protection. The efficacy of c-ABL inhibitors (Asciminib), serine-threonine kinases, S1P inhibitors, and C1P (ceramide-1-phosphate) to date are very limited.

Additionally, PI3K and mTOR pathway inhibitors are also under research. PI3K/mTOR pathways have important roles in cell proliferation and survival. Concomitant use of rapamycin and melatonin has been shown a role in preventing overactivation of the primordial follicles and preventing fertility [39,40].

A huge increase in research was conducted in recent years to discover new, more effective fertility preservation strategies but their efficacy is yet to be determined.

## RCT Results

Most studies have focused on the function of GnRHa combined with chemotherapy in patients with breast cancer, with few studies focusing on hematological malignancies and ovarian cancer.

### Breast cancer

In previous studies, differences have been observed in the patient populations and the definition of chemotherapy-induced POI. Most researchers have relied on amenorrhea; however, some have measured hormone levels. Furthermore, the follow-up period after chemotherapy has been different, ranging from 6 months to 5 years. Goserelin, triptorelin, and leuprolide acetate are the GnRH analogs that have been used.

### RCTs with goserelin

In 2008, Li et al. conducted the first study and included 31 patients with an average age of 40 years who received goserelin combined with chemotherapy in breast cancer patients. The evaluation period for chemotherapy-induced POI was 12 months after chemotherapy completion. They reported that the POI rate was statistically significantly lower in patients receiving goserelin (32.1%) compared with 53.1% (*p* = 0.027) [41].

Subsequently, in 2009, a prospective study that was conducted at a university hospital in Egypt was published. It involved 80 patients less than 40 years of age who were operated of breast cancer. The inclusion criterion for this study was an initial FSH level of <10 mU/mL. All patients received the FAC chemotherapy regimen, with goserelin therapy before chemotherapy. Hormone levels (FSH, LH, estradiol, progesterone, and prolactin) were measured in all patients until they resumed ovulation or menstruation. The researchers reported that POI was observed in 11.4% of patients 8 months after completing chemotherapy, compared with 33.3% in whom menstrual cycle resumed and 25.6% who exhibited normal ovarian function. In the control group (chemotherapy without GnRHaa), the menstrual cycle resumed in 33, 3% of individuals and normal ovarian activity resumed in 25, 6% of individuals [42].

In the ZIIP study, which was published in 2009, 256 patients receiving adjuvant therapy (chemotherapy or hormone therapy or both) after breast cancer surgery were included. Node-positive patients received the CMF chemotherapy regimen combined with hormone therapy (goserelin, goserelin plus tamoxifen, or tamoxifen alone over 2 years). The effects on ovarian function were monitored for 3 years after the study or 1 year after completing hormone therapy. Women with amenorrhea (64%) were significantly lower in the goserelin group compared with the control group (90%, *p* = 0.006) However, the use of menstruation as a surrogate of the ovarian function instead of the FSH, LH, estradiol, and AMH levels is a study limitation [43].

Five additional studies have been undertaken to examine the protective effect of goserelin on POI. The most recent one, i.e., (POEMS)/S0230, was conducted in 2015, with the final results being published in 2019. More than 200 patients with HR-negative breast cancer were randomly assigned to receive chemotherapy with or without goserelin. The chemotherapy regimen was based on cyclophosphamide combined with other chemotherapy agents. The primary objective was to determine the POI rate (with POI defined as the cessation of menstruation at least 6 months) and postmenopausal FSH levels 2 years after therapy. The POI rate was much lower in the goserelin group 8% and 22% in the chemotherapy-only group (odds ratio [OR], 0.30; 95% confidence interval [CI]: 0.09–0.97, *p* = 0.04). Furthermore, a higher percentage of women achieved pregnancy in the goserelin group (21%) compared with those in the chemotherapy group (11%, *p* = 0.03). Therefore, authors of the (POEMS)/S0230 study concluded that goserelin prevents early menopause and increases pregnancy likelihood after chemotherapy. After a 5-year follow-up period, the (POEMS)/SWOG S0230 study revealed a significantly higher incidence of pregnancies in the GnRH group (23.1% *vs*. 12.2%, *p* = 0.04). This is the only study that included pregnancy rate as a secondary endpoint. However, the inclusion of only patients with ER-negative disease and the inability to prove the safety and efficacy of GnRHs during chemotherapy for patients with ER-positive breast cancer are study limitations [44,45].

GBG 37 ZORO, a small-sample study involving 60 patients who received neoadjuvant sequential anthracycline and taxane-based therapy, with or without goserelin, revealed no statistically significant differences in the percentage of patients with amenorrhea between the goserelin and control groups 6 months after completing neoadjuvant therapy (70.0% with goserelin *vs*. 56.7% without goserelin; difference of 13.3%, *p* = 0.284) [46].

In the OPTION study, premenopausal patients with stage I–IIIB breast cancer with estrogen receptor-positive (ER+) and estrogen receptor-negative (ER−) tumors who were candidates for neoadjuvant or adjuvant chemotherapy were included. They underwent follow-up for 3 years after randomization, with monitoring of FSH levels. POI was defined as amenorrhea 12–24 months after randomization, with postmenopausal FSH levels of more than 25 IU/L. POI prevalence was 18.5% in patients randomized to receive goserelin, compared with 34.8% in the control group (*p* = 0.048). A subanalysis based on patient’s age revealed the protective role of goserelin in decreasing amenorrhea and POI in patients younger than 40 years of age (amenorrhea: 10.0% *vs*. 25.4%, *p* = 0.032; POI: 2.6% *vs*. 20.0%, *p* = 0.038); however, the effect was less evident and not statistically significant in patients more than 40 years of age [47,48].

Another study in which the sequential and concomitant administration of GnRH and chemotherapy were compared in women with ER+ breast cancer revealed that sequential administration was not inferior to concomitant administration in terms of early menopause rate, (cessation of menstruation lasting longer than 12 months after the last chemotherapy or GnRHa dose), with postmenopausal or unknown FSH and estradiol levels (simultaneous *vs*. sequential: OR, 1.01; 95% CI: 0.50–2.08, *p* = 0.969; OR, 1.13; 95% CI: 0.54–2.37, *p* = 0.737). Furthermore, it did not affect disease-free survival (*p* = 0.290) and overall survival (*p* = 0.514) [49].

A group of Chinese authors published in 2019 results of 98 breast cancer patients. Patients received chemotherapy with or without GnRHa regardless of hormone receptor positivity. The primary endpoint was POI at 1 year. The second endpoint was the change in AMH level during chemotherapy and the correlation between AMH and POI. POI rate was higher at 91.3% in the group with a low AMH baseline level of <1.1 ng/mL compared to 63.5% in the group with an AMH baseline level >1.1 ng/mL (*p* = 0.013). Results suggest that AMH level correlates with POI rate and that GnRHa may have a protective role, in both HR-positive and HR-negative patients, during chemotherapy (*p* = 0.013) [50].

Wang et al. conducted the study with newly diagnosed premenopausal breast cancer patients with stages I–III, who were assigned to receive (neo) adjuvant anthracyclines with or without taxanes-based chemotherapy. Patients were asked to choose to receive co-treatment with goserelin or not. It measured the levels of AMH, FSH, and E2 and the diameter of the antral follicle (AFC). The primary aim was recovery of AMH level 2 years after chemotherapy finished. The recovery rate of AMH was significantly higher in the goserelin group (46.5%) than in the control group (21.8%) (*p* < 0.002). The recovery rate of AFC and FSH were similar to AMH, significantly higher in the goserelin group (AFC: 44.1% *vs*. 19.6%, *p* < 0.002; FSH: 83.9% *vs*. 65.6%, *p* < 0.017). The results of recovery rate of E2 and menstruation were similar across groups (E2: 69.2% *vs*. 59.0%, *p* < 0.265; Menses: 69.9% *vs*. 66.0%, *p* < 0.739).

The limitation was the difference in baseline level of AMH because the age difference was evident between the group and 21.5% of enrolled patients had adjuvant endocrine therapy and were excluded from the analysis at 1 and 2 years after finished chemotherapy [51].

One of the largest studies conducted in China included 330 patients with stage I to III breast cancer. All the patients received neoadjuvant or adjuvant chemotherapy with or without GnRHa. The primary aim was the POI rate 1 year after chemotherapy. POI definition was AMH less than 0.5 ng/mL. The secondary endpoint was overall survival (OS) and tumor-free survival (TFS) The authors showed a significantly lower POI rate in the GnRHa group compared to chemotherapy only (10.3% *vs*. 44.5%, *p* < 0.001) and the AMH recovery rate was better in GnRHa group (15 of 25 *vs*. 6 of 44; *p* < 0.001). After a median follow-up of 49 months, there are no significant differences in 4-year OS and TFS between the 2 groups. Subgroup analysis showed that patients less than 35 years old had longer TFS in the GnRHa group compared to chemotherapy chemotherapy-only group (93% *vs*. 62%; *p* = 0.004). The limitation of this study was that the study did not have fertility as the end point of the study because the authors did not recommend pregnancy in the first 3 years after diagnosis because of the high risk of relapse at that time [52]. Table 1 summarizes the results of all studies involving goserelin.

**Table 1:** RCTs investigating ovarian suppression in patients with breast cancer who received goserelin during chemotherapy

Authors/year	POI	Time when POI evaluated months	No. of patients	Results	Conclusion
Li et al. 2008 [41]	Amenorrhea	12	31 CT + GnRHa	POI rate	Prevention of POI
			32 CT	32.1% *vs*. 53.1% (*p* = −0.027)	
Badawy et al. 2009 [42]	Amenorrhea without ovulation	8	39 CT + GnRHa	POI rate	Prevention of POI
				11.4% *vs*. 66.6%	
			39 CT	(*p* < 0.001)	
Sverrisdottir et al. 2009 [43]	Amenorrhea	36	51 CT + GnRHa	POI rate	Prevention of POI
			43 CT	64% *vs*. 90% (*p* = 0.006)	
Moore et al. 2015 2019 [44,45]	Amenorrhea and FSH level postmenopausal	24	115 CT + GnRHa	POI rate	Prevention of POI and pregnancies
				8% *vs*. 22%	
			113 CT	(*p* = 0.04)	
				Pregnancies: 23 *vs*. 13	No impact on DFS an OS
				(*p* = 0.04)	
				5-yDFS	
				88.1% *vs*. 78.6%	
				(*p* = 0.09)	
				5-y OS	
				91.7% *vs*. 83.1% (*p* = 0.06)	
Leonard et al. 2017 [48]	Amenorrhea and FSH and E2 level postmenopausal	24	103 CT + GnRHa	POI rate:	Prevention of POI
				18.5% *vs*. 34.8%	
			118 CT	(*p* = 0.048)	
Zhang et al. 2018 [49]	Amenorrhea and FSH and E2 level postmenopausal	36	108 CT + GnRHa	POI rate: 23.1% *vs*. 22.8%	No protection of POI
			108 CT	(*p* = 0.969)	
Zhong et al. 2019 [50]	Amenorrhea AMH level	12	51 CT + GnRHa	POI rate	Prevention of POI
			45 CT	44.7% *vs*. 80.6%; (*p* = 0.002).	
Wang et al. 2021 [51]	Amenorrhea AMH level	24	73 CT + GnRHa	POI rate	Prevention of POI
				21.8% *vs*. 46.5%	
			76 CT	(*p* < 0.002)	
Zhong et al. 2022 [52]	Amenorrhea AMH level	12	165 CT + GnRHa	POI rate 10.3% *vs*. 44.5%	Prevention of POI
			165 CT	(*p* < 0.001)	No impact of OS and TFS

Note: CT: chemotherapy, POI: premature ovarian insufficiency, FSH: follicle-stimulating hormone, LH: Luteinizing hormone, TFS: tumor-free survival.

### RCTs with triptorelin and leuprolide acetate

Five randomized studies have evaluated the effects of triptorelin. In 2011, the initial results of the PROMISE-GIM6 study were published by a group of Italian authors [53]. This phase 3 superiority study was conducted in 16 centers in Italy and included 281 patients with stage I–III breast cancer who were candidates for neoadjuvant or adjuvant therapy. The patients received the anthracycline, anthracycline, and taxane or CMF chemotherapy regimen. Most patients (80.4%) were diagnosed with hormone receptor-positive breast cancer. Early menopause, defined as the absence of menstruation for 12 months after chemotherapy and postmenopausal FSH and estradiol levels, was observed in 25.9% of patients who only received chemotherapy and in 8.9% of patients who received triptorelin. The absolute decrease of 17% in the rate of early menopause (95% CI: −26% to −7.9%; *p* < 0.001) provides evidence that combining the GnRHa triptorelin is vital for protecting the menstrual cycle. This was maintained in the long-term, with a higher number of pregnancies observed in patients who received GnRHa during chemotherapy [54]. Final results after 12.4 years of follow-up revealed no difference in the 12-year disease-free survival between the GnRHa group (65.7% [95% CI: 57.0%–73.1%]) and control group (69.2% [95% CI: 60.3%–76.5%], HR = 1.16). Similarly, no significant difference was observed in the 12-year overall survival between the GnRHa group (81.2% [95% CI: 73.6%–86.8%]) and control group (81.3% [95% CI: 73.1%–87.2%], HR = 1.17). In update analysis it was added a small subgroup of patients with germline BRCA1/2 pathogenic variants, 5 in BRCA1 (3 received GnRHa) and 5 in BRCA2 (1 received GnRHa). The incidence of POI was 0% (GnRHa arm) and 33% (control arms). Because of the small number of patients in this subgroup, this is only a descriptive analysis.

Authors conclude that the final results of the PROMISE-GIM6 trial support the use of GnRHa during chemotherapy as a strategy to protect ovarian function, including in patients with hormone receptor–positive disease [53,54].

In Table 2, it is shown that two studies failed to demonstrate the protective effect of triptorelin on ovarian function. In a study involving 100 patients with ER-breast cancer, no difference was observed in menstruation resumption rates between the GnRH and control groups (both 80%, *p* = 1.00) at 12 months after completing cyclophosphamide-based chemotherapy [55]. In another study involving 124 patients who received anthracycline-based chemotherapy or anthracycline and taxane, the patients were stratified based on age. The amenorrhea rate was comparable between the groups regardless of the patient’s age, chemotherapy regimen, or ER status [56].

**Table 2:** RCTs investigating ovarian suppression with triptorelin during chemotherapy in patients with breast cancer

Authors/year	POI	Time when POI evaluated months	No. of patients	Results	Conclusion
Lambertini et al. 2022 [54]	Amenorrhea and postmenopausal levels of FSH and E2	12	148 CT + GnRHa	POI rate	Prevention of POI
				8.9% *vs*. 25.9%	
			133 CT	(*p* < 0.001)	No impact on DFS and OS
Elgindy et al. 2013 [55]	Amenorrhea	12	50 CT + GnRHa	POI rate	No prevention of POI
				20% *vs*. 20%	
			50 CT	(*p* = 1.00)	
Munster et al. 2012 [56]	Amenorrhea	24	27 CT + GnRHa	POI rate	No prevention of POI
				15% *vs*. 14%	
			22 CT	(*p* = 0.32)	
				Pregnancies: 0 *vs*. 2	
Jiang et al. 2013 [57]	Amenorrhea	--	10 CT + GnRHa	POI rate:	Prevention of POI
				10.0% *vs*. 45.5%	
			11 CT	(*p* = 0.05)	
Karimi-Zarchi et al. 2014 [58]	Amenorrhea	6	21 CT + GnRHa	POI rate	Prevention of POI
				9.5% *vs*. 66.7%	
			21 CT	(*p* < 0.001)	

Note: CT: chemotherapy, POI: premature ovarian insufficiency, FSH: follicle-stimulating hormone, E2: estradiol, DFS: disease-free survival. OS: overall survival.

In contrast, in other studies, the ovarian protective role of triptorelin has been demonstrated, including the studies by Jiang et al. and Karimi-Zarchi et al. Jiang et al. reported that the POI rate was 10% in the GnRH group compared with 45.5% in the chemotherapy alone group (*p* = 0.05). Furthermore, Karimi-Zarchi et al. reported that the POI rate was 9.5% in the GnRha group compared with 66.7% in the group without triptorelin (*p* < 0.001). Results are shown in Table 2 [57,58].

To date, only one prospective study has investigated the role of leuprolide acetate. It included 220 patients who received the cyclophosphamide–doxorubicin regimen with or without a GnRHa. Premenopausal FSH and estradiol levels within 1 year after completing chemotherapy and recovery of menstrual cycle were considered ovarian preservation. At the end of the follow-up period, the menstrual cycle resumed in 27/94 patients in the chemotherapy-only group and 15/89 patients in the GnRHa group; furthermore, premenopausal FSH and estradiol levels were restored in 7 patients in the chemotherapy-only group and 14 in GnRHa group. Therefore, the concomitant administration of leuprolide acetate with chemotherapy decreases the risk of premature menopause in premenopausal patients with breast cancer [59].

### Hematological and other malignancies

Because the protocols used for treating lymphomas include chemotherapy agents that significantly affect POI, several studies have been conducted in this area. However, the total number of patients has been extremely small, i.e., approximately 150; therefore, the data should be analyzed carefully. All four studies involving patients with lymphomas failed to demonstrate the protective role of GnRHa on ovarian function. The first study was published in 1987 by Waxman et al.; they investigated buserelin as a GnRHa in 30 men and 18 women with Hodgkin’s lymphoma (HL); these patients were followed up for 3 years after treatment. Buserelin did not exert a protective effect on premature menopause [8].

Subsequently, the protective function of triptorelin was investigated in 29 women with HL who received the following regimens: ABVD, cyclophosphamide/vincristine/procarbazine/prednisone, or cisplatin/cytarabine/dexametha-sone. In addition to monitoring the menstrual cycle, FSH, LH, and AMH levels were also measured. The time elapsed since chemotherapy was the only prognostic marker for ovarian function. Triptorelin did not exhibit a protective effect, even though it delayed ovarian failure in breast cancer patients. Similar results were observed for the 1-year POI rate in the GnRH group compared with the chemotherapy group in another study investigating the protective effect of triptorelin (20% *vs*. 19%; *p* = 1.00) [60,61].

Nevertheless, these results should be analyzed carefully because differences exist between premenopausal patients with lymphoma and those with breast cancer. Patients with lymphoma are generally younger during cancer diagnosis and receive chemotherapy regimens that range from a very low to extremely high POI risk. Furthermore, the number of patients in the studies was small, with a total of only 154 patients across all studies.

To date, only one randomized prospective study has investigated the protective effect of GnRH in women with ovarian cancer who received chemotherapy, i.e., multi-drug regimens of bleomycin/etoposide/cisplatin/taxol/carboplatin or taxol/cisplatin or the vincristine/actinomycin/cyclophos-phamide regimen. Diphereline 3.75 mg was used as the GnRH agonist. In the LHRHa group, the menstrual cycle resumed in most patients within 2–4 months, indicating that disheveling exerts a positive effect on fertility preservation in patients with ovarian cancer [62].

### Meta-analysis of RCTs

Since 2010, over 20 meta-analyses of RCTs have investigated the protective effect of GnRH on ovarian function and fertility. Most studies have focused on patients with breast cancer, hematological malignancies, and autoimmune diseases. Table 3 summarizes the results of the meta-analyses that exclusively focused on patients with breast cancer. Notably, different definitions were used for POI in these studies. Furthermore, the follow-up duration was different.

**Table 3:** Overview of the meta-analyses investigating the protective effect of GnRHa in chemotherapy for breast cancer

Authors	Year	Type of cancer	No. of studies	No. of patients	Results	Conclusion
Yang et al. [63]	2013	Breast cancer	5	528	POI: RR 0.40 (95% CI 0.21–0.75)	Protective for POI, not for pregnancy
					Pregnancies: RR 0.96 (95% CI 0.20–4.56)	
Wang et al. [64]	2013	Breast cancer	7	677	POI: OR 2.83 (95% CI 1.52–5.25)	Protective for POI
Shen et al. [65]	2015	Breast cancer	11	1062	POI 30% *vs*. 45%; OR 2.57 (95% CI 1.65–4.01)	Protective for POI and pregnancy
					Pregnancies: 26 (9%) *vs*. 16 (6%); OR 1.77 (95% CI 0.92–3.40)	
Lambertini et al. [66]	2015	Breast cancer	12	1231	POI: 19% *vs*. 34%; OR 0.36 (95% CI 0.23–0.57) Pregnancies: 9% *vs*. 6%; OR 1.83 (95% CI 1.02–3.28)	Protective for POI and pregnancy
					DFS: HR 1.00 (95% CI 0.49–2.04), *p* = 0.939)	No impact on DFS
Munhoz et al. [67]	2016	Breast cancer	7	856	POI at 6 months: 26% *vs*. 43%; OR 2.41 (95% CI 1.40–4.15)	Protective for POI and pregnancy
					Pregnancies: OR 1.85 (95% CI 1.02–3.36)	
Silva et al. [68]	2016	Breast cancer	7	1002	POI: 26% *vs*. 39%; OR 2.03 (95% CI 1.18–3.47)	Protective for POI
Bai et al. [71]	2017		15	1540	POI: 23% *vs*. 43%; OR 1.36 (95% CI 1.19–1.56)	Protective for POI and pregnancy
					Pregnancies: 7% *vs*. 4%; OR 1.90 (95% CI 1.06–3.41)	
Lambertini et al. [9]	2018	Breast cancer	5	873	POI: 14% *vs*. 31%; OR 0.38 (95% CI 0.26–0.57)	Protective for POI and pregnancy
					Pregnancies: 10% *vs*. 6%; IRR 1.83 (95% CI 1.06–3.15)	
					DFS: HR 1.01 (95% CI, 0.72 to 1.42; *p* = 0.999)	No impact on DFS and OS
					OS: HR 0.67 (95% CI, 0.42 to 1.06; *p* = 0.083)	

Note: POI: premature ovarian insufficiency, RR: response rate, DFS: disease-free survival, OS: overall survival interval.

Yang et al. included five RCTs published before 2012 (528 patients) which were compared impact of GnRHa plus chemotherapy compared to chemotherapy alone on POI in premenopausal breast cancer patients. As it is mentioned in Table 3, authors conclude that GnRHa are protective for POI and not for pregnancy [63].

Wang et al. with a higher number of patients (677) also conclude that the use of GnRHa with chemotherapy may be protective for POI and provide short term return of menstruation pregnancy [64]. The benefit of GnRHa was more prominent in studies including patients with primarily breast cancer [9,65–68]. In general, only two meta-analyses failed to demonstrate the role of GnRHa on ovarian protection [69,70].

Over the past 5 years, after many RCTs have been conducted, not only in breast cancer but also in hematological malignancies, ovarian cancer, and autoimmune diseases, many significant meta-analyses with a larger number of patients have been published. Chemotherapy has efficacy in certain autoimmune diseases because cytotoxic agents decrease cell proliferation and reduce the level of products that cause inflammations like cytokines. All these meta-analyses involving smaller patient populations published before 2018, except for one, revealed the protective role of GnRHa on ovarian function [72,73].

In Table 4, it is shown meta analysis of Hickman et al. with 10 RTCs met inclusion criteria with patients with lymphoma, ovarian cancer and breast cancer. From ten RCTs 8 studies support the use of GnRHa with chemotherapy *vs*. chemotherapy alone for preservation of ovarian function (POI rate 14% *vs*. 31% (OR 0.38, 95% CI 0.26–0.57)) [74]. In 1208 patients with lymphoma and breast cancer, Senra et al. found also protective role for POI with GnRHa with chemotherapy but not for pregnancy [75]. In 2019, three more meta analysis was done and confirmed protective role of GnRHa on ovarian function. The meta analysis of Sofiyeva et al. included patients with autoimmune diseases and statistical significance difference with GnRHa cotreatment was consistent in all subpopulation of patients including breast cancers, hematological and autoimmune diseases [76–78].

**Table 4:** Overview of the largest meta-analyses of RCTs investigating the protective effect of GnRH in breast cancer, hematological malignancies, ovarian cancer, and autoimmune diseases

Authors	Year	Type of disease	No. of studies	No. of patients	Results	Conclusion
Hickman et al. [74]	2018	Lymphoma, ovarian cancer, breast cancer	10	1051	POI: 14% *vs*. 31% (OR 0.38, 95% CI 0.26–0.57)	Protection for POI
Senra et al. [75]	2018	Lymphoma, breast cancer	13	1208	POI (RR, 0.60; 95% CI, 0.45–0.79)	Protection for POI, not for pregnancy
					Pregnancy	
					(RR, 1.43; 95% CI, 1.01–2.02)	
Zheng et al. [76]	2019	Lymphoma, breast cancer	12	1413	POI (RR = 0.47, 95% CI = 0.31–0.71, *p* = 0.0004).	Protection for POI, not for pregnancy
					Pregnancy (RR = 1.40, 95% CI = 0.98–1.98, *p* = 0.06)	No impact on DFS and OS
					DFS (RR = 1.04, 95% CI = 0.95–1.13, *p* = 0.40)	
					OS (RR = 1.02, 95% CI = 0.90–1.16, *p* = 0.72)	
Chen et al. [77]	2019	Lymphoma, ovarian cancer, breast cancer	12	1369	POI 10.7% *vs*. 25.3% (RR 0.44, 95% CI 0.31 to 0.61)	Protection for POI, not for pregnancy
					Pregnancy 9% *vs*. 6.3% (RR 1.59, 95% CI 0.93 to 2.70)	
Sofiyeva et al. [78]	2019	Autoimmune diseases, breast cancer, lymphoma	18	1043	POI (RR: 1.38; 95% CI: 1.18–1.63)	Protection for POI

Note: POI: premature ovarian insufficiency, RR: response rate, DFS: disease-free survival, OS: overall survival.

All but two meta-analyses revealed the protection strategy in decreasing chemotherapy-induced POI risk in premenopausal patients with cancer [69,70]. The effect was noted to be more pronounced in meta-analyses that included only patients with breast cancer compared with those that included patients with hematological malignancies.

### Observations of the available clinical data

In the last more than three decades of very active research in the field of oncofertility, data on administering GnRHa during chemotherapy to protect ovarian function and fertility, in breast cancer, are mainly consistent while the results of the study in premenopausal patients with hematological malignancies are negative. There are some explanations for this discrepancy. First, there is a big difference in the number of patients in clinical trials, only about 150 patients with lymphoma and about 2000 patients with breast cancer were included in studies. Patients with lymphoma are diagnosed at a younger age than breast cancer patients and have higher ovarian reserve and acute POI is evident only after high-risk gonadotoxicity therapy. In breast cancer patients, the benefit of using GnRHa becomes evident much earlier because of lower ovarian reserve and has been observed in different types of chemotherapy agents [9].

The second important thing is, to date, that there is no unique definition of chemotherapy-induced POI. In the majority of studies, POI was defined as amenorrhea but it is not a perfect surrogate marker of the gonadotoxicity. Experts agree that the definition of chemotherapy-induced POI includes amenorrhea for ≥2 years and a post-menopausal hormonal profile. Only a few trials evaluated AMH and hormonal profiles [48,51,55,61,79].

The third thing is the trials did not have an aim rate of post-treatment pregnancies, in inclusion criteria did not include pregnancy desire, and the follow-up period was not enough long to assess that important outcome. A small number of patients achieved pregnancy after chemotherapy for breast cancer, but the number is higher in patients who were protected with GnRHa [9,67,71,75].

## Conclusion

Oncofertility is an extremely significant topic that is gaining recognition owing to surplus evidence indicating that fertility preservation does not affect the treatment outcomes of patients with cancer and that it significantly contributes to preserving the patient’s quality of life by facilitating their desire to start a family. Even if no desire for pregnancy exists, preserving ovarian function is vital owing to the symptoms and overall effect that premature menopause causes on women’s health [10,80].

However, conflicting evidence exists regarding the recommendation of GnRHa for fertility preservation. Current guidelines from the ASCO encourage sperm, oocyte, and embryo cryopreservation as a standard of care practice and offering GnRHa to patients only when proven fertility preservation methods are not feasible; furthermore, it should not be used to replace this proven fertility preservation method. The coadministration of a GnRHa with chemotherapy can be used along with the proven fertility preservation methods [81].

The ESMO Clinical Practice Guidelines provide a similar conclusion that sperm, oocyte, or embryo cryopreservation is the preferred option and that GnRHa during chemotherapy may be offered as an additional option after cryopreservation strategies or when they are not feasible [10]. The PREFER study revealed that less than 20% of women less than 40 years of age accepted cryopreservation as a fertility preservation method, whereas more than 90% accepted the concurrent use of GnRH during chemotherapy to protect ovarian function. Furthermore, 90.6% of women over 40 years of age, who are normally not candidates for cryopreservation, accepted the administration of GnRHa with chemotherapy to protect ovarian function and mitigate the adverse effects associated with POI. Importantly, in patients interested in fertility preservation, the administration of GnRHa should not be considered an alternative to cryopreservation methods [82].

Notably, most randomized studies on the effectiveness of GnRHa during chemotherapy in preventing POI have been conducted in women with breast cancer, with a considerably small number of studies on approximately 150 patients with hematological malignancies. Most RCTs on breast cancer have revealed a decrease in the risk of treatment-induced POI, regardless of the hormone receptor status. However, the short follow-up period has been a recurring limitation of most studies. On the other hand, studies on hematological malignancies have yielded negative results; however, the findings must be interpreted with caution owing to several limitations.

Moreover, at present, we are witnessing the widespread use of ICIs and PARP inhibitors for treating early breast cancer. However, limited data are available on their effects on ovarian dysfunction; therefore, future research should be focused on that direction [83]. Another important aspect is that for a subset of patients with early breast cancer, de-escalation of chemotherapy can be considered an option with similar treatment outcomes but with a lower rate of therapy-induced amenorrhea. De-escalation of therapy is also being investigated in other cancer types. Therefore, de-escalation of chemotherapy represents an important approach to protecting ovarian function.

Future research should not only focus on treatment efficacy but also simultaneously monitor ovarian reserve and the effect of new therapies on POI. Furthermore, additional studies are warranted to follow the long-term effect of GnRHa, including post-treatment pregnancies and age at menopause, from existing RCTs and investigate more sensitive biomarkers for ovarian reserve, including AMH levels and antral follicle count [10,83]. Also, future clinical studies can focus on some other alternative pharmacological methods for fertility protection and need to better understand mechanisms of action in how GnRHa protects against ovarian failure. To acquire a more robust conclusion we need large prospective randomized studies on these topics to evaluate the safety and efficacy of fertility-preserving strategies in cancer patients.

## Data Availability

Not applicable.
